# Gene-Based Therapeutics for Parkinson’s Disease

**DOI:** 10.3390/biomedicines10081790

**Published:** 2022-07-26

**Authors:** Karim E. Shalaby, Omar M. A. El-Agnaf

**Affiliations:** 1 Biological and Biomedical Sciences Division, College of Health & Life Sciences (CHLS), Hamad Bin Khalifa University (HBKU), Qatar Foundation, Doha 34110, Qatar; kshalaby@hbku.edu.qa; 2 Neurological Disorders Research Center, Qatar Biomedical Research Institute (QBRI), Hamad Bin Khalifa University (HBKU), Qatar Foundation, Doha 34110, Qatar

**Keywords:** neurodegeneration, Parkinson’s disease, gene therapy

## Abstract

Parkinson’s disease (PD) is a complex multifactorial disorder that is not yet fully surmised, and it is only when such a disease is tackled on multiple levels simultaneously that we should expect to see fruitful results. Gene therapy is a modern medical practice that theoretically and, so far, practically, has demonstrated its capability in joining the battle against PD and other complex disorders on most if not all fronts. This review discusses how gene therapy can efficiently replace current forms of therapy such as drugs, personalized medicine or invasive surgery. Furthermore, we discuss the importance of enhancing delivery techniques to increase the level of transduction and control of gene expression or tissue specificity. Importantly, the results of current trials establish the safety, efficacy and applicability of gene therapy for PD. Gene therapy’s variety of potential in interfering with PD’s pathology by improving basal ganglial circuitry, enhancing dopamine synthesis, delivering neuroprotection or preventing neurodegeneration may one day achieve symptomatic benefit, disease modification and eradication.

## 1. Features of Parkinson’s Disease: From Theory to Therapy

### 1.1. Prevalence, Biology and Pathophysiology

Parkinson’s disease (PD) is the second most common neurodegenerative disorder with a prevalence that increases steadily with age, affecting 0.3–0.4% of people between 65 and 70 years and peaking to almost 1.2–1.7% between 85 and 89 years globally [1,2]. The total annual cost of PD to patients, their families and healthcare is estimated to be around USD 52 billion in the United States alone, a figure expected to grow substantially in the next few decades with the rapidly growing size of the elderly population [3,4]. The symptoms of the disease include a range of motor deficits including tremors, rigidity and bradykinesia [5]. Patients frequently experience additional non-motor symptoms that involve cognitive and behavioral complications as the disease progresses [6].

**Figure 1:**
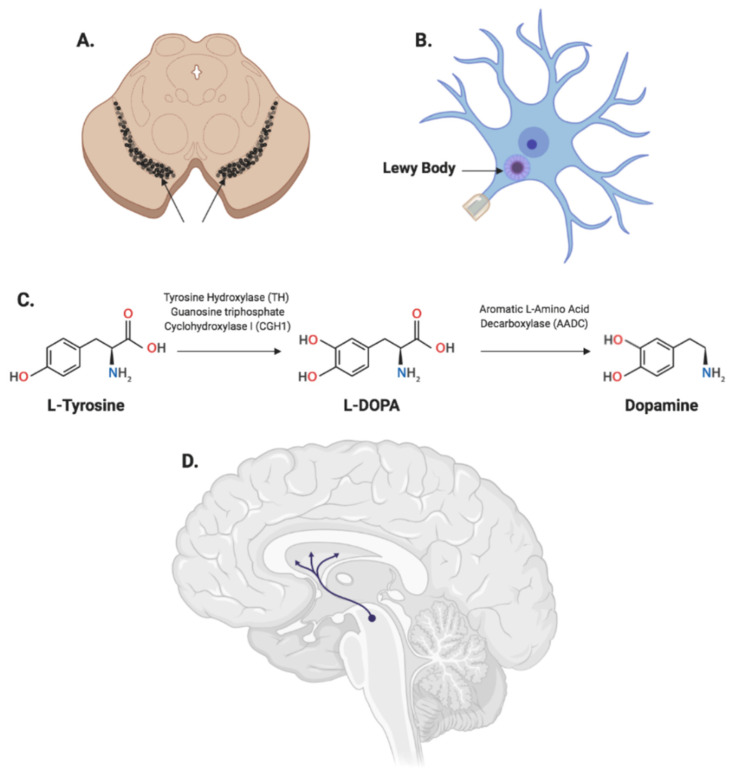
Neuro-pathophysiology of Parkinson’s disease. (**A**) Substantia nigra (black substance): patients in late stages of Parkinson’s disease (PD) lose more than half of the dopaminergic neurons in this region. (**B**) Intracellular Lewy body inclusions in dopaminergic neurons, a characteristic hallmark sign of PD. (**C**) Dopamine is obtained from diet-derived L-tyrosine; L-tyrosine is catalyzed to produce L-DOPA by tyrosine hydroxylase (TH) and guanosine triphosphate cyclohydroxylase I (CGH1), which is in turn decarboxylated to dopamine by aromatic l-amino acid decarboxylase (AADC). (**D**) Nigrostriatal pathway: connects nigral neurons to the striatum and aids with dopamine transport within the basal ganglia.

PD is a disease of the basal ganglia, a group of structures involved primarily in the facilitation of movement, among other functions. The neuro-pathophysiology of the disease is characterized by the progressive degeneration of dopaminergic neurons in the mid-brain’s substantia nigra pars compacta (SNc) (Figure 1A,D). This results in a reduction in the levels of the neurotransmitter dopamine being produced and projected to the striatum through the nigrostriatal pathway (Figure 1D). The plummeting levels of dopamine in a PD patient’s brain trigger physiological imbalances in the dopamine signaling pathways throughout the basal ganglia. Particularly, the over-activity of the subthalamic nucleus (STN), normally inhibited in a healthy brain, leads to the expression of motor symptoms.

It is also important to note that PD is widely considered to be a form of proteinopathy. The progression of neuronal loss is more specifically associated with an aberrant accumulation of intracytoplasmic inclusions of the α-synuclein protein in Lewy bodies (Figure 1B) within the vicinity of dopaminergic neurons [7]. α-Synuclein is a small protein (140 amino acids), abundantly expressed in the brain, whose exact function is unclear, but it is known to play a role in the regulation of dopamine release through synaptic vesicle clustering [8,9]. The α-synuclein pathology is thought to spread to other neurons; essentially, extracellular α-synuclein enters neighboring neurons through endocytosis, leading to intracellular inclusions and aggregates [10]. The role of these aggregates in neurotoxicity is still a subject of controversy; however, they are present in a number of other neurodegenerative disorders as well, most notably in dementia with Lewy bodies, albeit with varying pathological processes and localization patterns across the nervous system [11]. Moreover, the increasing accumulation of these inclusions is associated with the development of non-motor symptoms in PD patients [12].

It is suggested that α-synuclein’s secretion by neurons leads to propagation of the Lewy body pathology, thereby extending the reach of neurodegeneration. Previous post mortem studies of PD patients who had fetal nigral cells transplanted into their striatum showed that grafted neurons experience a host-to-graft Lewy body pathology to the same extent as SNc neurons in cases of PD [13,14,15,16]. Whether spreading is by a prion-like process, or it is simply the debris of cells claimed by a Lewy body pathology, it is established that α-synuclein is present in the extracellular matrix and cerebrospinal fluid [17]. Hence, it is as necessary to target extracellular α-synuclein as it is to target intracellular inclusions in order to halt disease progression. It was shown in cultured neuronal cells that the accumulation of α-synuclein may be specifically caused by the impairment of chaperone-mediated autophagy (CMA), a process of degradation which occurs in lysosomes [18]. Injections of AAV-Lamp2a, the protein that induces CMA, reduce α-synuclein levels, preserving nigral cells, nigrostriatal terminals and dopamine levels in the substantia nigra of preclinical models [19]. Furthermore, mechanisms of eliminating extracellular α-synuclein levels need to be considered for the treatment of PD, and it has been demonstrated through the delivery of cleavage enzymes such as calpains and neurosins [20,21].

### 1.2. Genetic Architecture of PD

PD is likely to be affected by both genetic and environmental factors [22]. In some cases, highly penetrant variants will not result in disease manifestation. In addition, patients with the same variant and within families can differ in age of onset and disease severity and progression [23]. Significant progress has been made in the identification of rare genetic mutations that contribute to at least 30% of familial and 3–5% of sporadic PD [24], with disease-causing mutations in more than 20 genes reported to date [23].

A missense mutation in SNCA, the gene encoding α-synuclein, was discovered in early family studies of PD [25]. Later, point mutations in SNCA (A53T [26,27], A30P [28], E46K [29], G51D [30], H50Q [31], A53V [32], A53E [33] and A30G [34]), as well as duplication [35], triplication [36] and promoter polymorphisms, have been associated with inducing the abnormal formation of Lewy bodies in familial and sporadic forms of PD [37,38,39]. The presence of copy number variants in patients of PD confirms an undeniably strong link between the level of expression of α-synuclein and the development of PD. However, such mutations account only for a small proportion of PD cases. Common variability in the *SNCA* locus was also associated with PD risk and the age of onset of sporadic PD, as confirmed by genome-wide association studies (GWASs) [40,41,42,43,44].

Discovered in 2004, LRRK2 has also been identified as one of the greatest genetic contributors to familial and sporadic PD, including missense variants of high penetrance [45] and moderate penetrance [46] and risk factors identified in GWASs [14,47]. The LRRK2 protein contains both kinase and Rab GTPase enzymatic activities thought to play a role in the secretory pathway, adult neurogenesis, remodeling of the cytoskeletal architecture and dopaminergic signaling [48]. Notably, humans with partial loss of function in LRRK2 lack any phenotype manifestation, suggesting that knocking out the harmful allele could prove to be a safe therapeutic strategy [24]. Other examples of pathogenic variants include mutations in eIF4G1 that have been suggested to play an important role in the disease pathogenicity. eIF4G1 is known to regulate autophagy and protein translation through mTOR-dependent nutrient sensing [49]. Although rare, pathogenic mutations in this gene have been associated with Lewy body pathology in many populations [50].

Moreover, variants in PARK7 (DJ-1), PRKN (PARK2) and PINK1 have been associated with mitochondrial and mitophagy function [23]. More recently, heterozygous loss-of-function mutations in GBA, the causative gene for Gaucher’s disease, have also been associated with a >5-fold increased risk of PD and the accumulation of Lewy bodies [51,52]. GBA encodes glucocerebrosidase (GCase), a protein involved in lysosomal degradation. In Gaucher’s disease, a build-up of fatty acids is caused by hampered lysosomal degradation, leading to enlarged tissues. Similarly, it is suggested that lower levels of GCase may weaken the helical binding of α-synuclein to lipid membranes, causing it to accumulate [53]. Moreover, evidence from animal PD models suggests that decreased GCase activity affects degradative pathways and increases the risk of developing PD [54]. Likewise, variants in LRRK2 and VPS35 are likely involved in lysosomal and trafficking pathways [23]. Thus, processes that affect cells’ ability to degrade α-synuclein, such as lysosomal dysfunction, are rising as plausible primary targets for PD therapy. How these genetic mutations contribute to cell loss and the formation of Lewy bodies might not yet be fully understood, but they may help define the critical molecular pathways that may be affected and play a role in neurodegeneration in PD.

Developing a therapy for PD patients would thus need to consider the different disease mechanisms responsible, based on the patients’ display of genetics and biomarkers. Categorizing sporadic cases of PD would be more complex than familial cases (with mutations in SNCA or LRRK2). First, describing PD as sporadic, or to arise with an unknown cause, would be an oversimplification. Currently, GWASs facilitated by parallel sequencing methods that are capable of the enriched screening of entire genomes for risk alleles have revealed a far wider genetic insight towards a large proportion of PD cases than was ever possible through early genetic linkage or array-based assays [23]. A number of the GWAS-identified loci are pleomorphic risk loci that are close to “familial” PD genes such as SNCA, LRRK2, GBA and VPS13C [55]. Others lie in close proximity to genes responsible for frontotemporal dementia and amyotrophic lateral sclerosis (MAPT, GRN, NEK1) [56], and Crohn’s disease and Blau syndrome (NOD1) [57]. Furthermore, it is estimated that 22% of PD is caused by common genetic variability, of which GWASs so far explain only a fraction, and thus more mutations continue to be discovered [23].

### 1.3. Current Treatment Strategies

**Figure 2:**
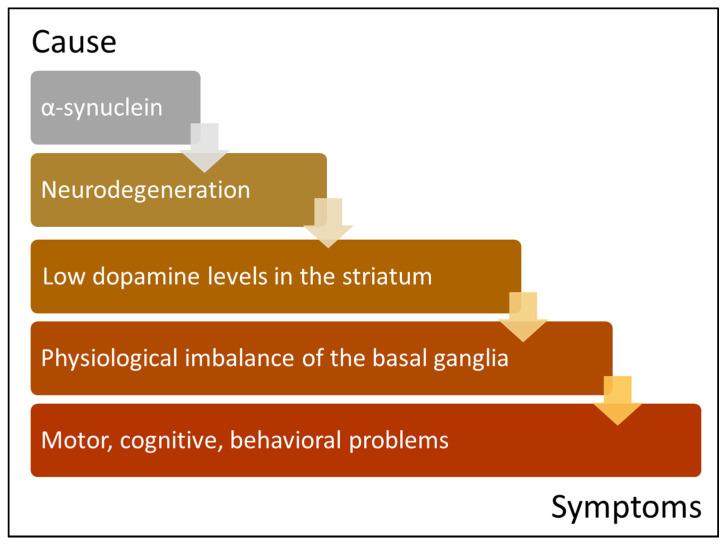
Hierarchy of Parkinson’s disease pathogenesis. This figure illustrates the multiple levels where gene therapy is capable of intervention in the treatment of Parkinson’s disease. From bottom upwards: the motor and non-motor symptoms of Parkinson’s disease are caused by the over-activity of the subthalamic nucleus in the basal ganglia due to a reduction in dopamine levels caused by the death of dopaminergic neurons. This cascade of events is thought to be triggered by the α-synuclein pathology in those neurons. Left: examples of gene therapy trials discussed in this review (GAD, AADC, TH, GDNF, RNAi); and right: examples of current therapeutic strategies aimed at each level).

According to the current understanding of PD, several different therapeutic approaches were designed to address each of the pathological processes involved (Figure 2). Current standards of care, however, have a short duration of effect and can exacerbate the disease’s cognitive and motor deficits as their side effects become more pronounced with their prolonged use. Examples of such established therapies include the administration of oral medications such as levodopa (L-DOPA), a dopamine precursor which increases dopamine levels in the brain. Conversely, creatine and Co-enzyme Q are aimed upstream towards protecting dopaminergic neurons from degeneration through enhancing the respiratory chain complex and ATP synthesis. In addition, there are surgical interventions such as deep-brain stimulation (DBS), which involves the insertion of electrodes into the basal ganglia to increase the inhibitory tone of the STN. These therapies offer mere symptomatic relief that becomes less effective as the disease progresses and more neurons are lost [58]. A lot of promise lies in cell-replacement therapies, particularly those comprising dopaminergic neurons derived from human-induced pluripotent stem cells, which are likely to be at the forefront of the battle against PD [59]. Evidence of host-to-graft spread of pathology, however, may limit the efficacy of such transplants on the long term, especially in younger patients with an earlier disease onset or diagnosis [13,14,15,16]. Strategies such as avoiding autologous grafts, or engineering donor cells to enhance their resistance to pathology will need to be evaluated to improve stem-cell-based technologies [59]. In most cases when PD is diagnosed, progressive neurodegeneration is well advanced, which gives clinicians little capacity for treatment. This necessitates the call for developing biomarkers to enable the identification of the earliest stages of the disease. In context with this projection, a different venue of immunotherapy aims to develop specific antibodies that block α-synuclein protein aggregation. Thus far, such strategies are being developed, such as antibodies optimized to cross the blood–brain barrier (BBB) and bind to α-synuclein proteins around and within neuronal cells [60,61]. The specificity of such molecules is potentially suited for use in imaging α-synuclein as a biomarker for the detection of early stages of its aggregation. There are several proposed pathways underlying the propagation of α-synuclein to further neuronal cells once found in extracellular space, including through cell-to-cell transmission [62]. Approaches designed to achieve extracellular α-synuclein clearance or limit its endocytosis via α-synuclein receptor blocking may delay the progression of the Lewy body pathology [62]. The main reason behind the failure of current treatments, however, is that they do not tackle the underlying cause of the disease, but rather target pathways that slow down its progression. The need to shift towards therapies capable of disease modification is clear and is what gene therapy hopes to achieve in the near future. We discuss here the various approaches of PD gene therapy, which address the shortcomings of current drug treatments and explore the potential of neuroprotection and precision medicine [63].

## 2. Gene Therapy for PD: Principles and Practicalities

The high potential of gene therapy gives immense hope for curing thousands of diseases with limited treatment options, such as cancer and monogenic diseases [64,65]. Gene therapy is either carried out ex vivo, involving the genetic modification of cultured cells and transplanting them into the patient, or in vivo, involving the use of vectors to deliver nucleic acids (DNA, RNA, etc.) or genome-modifying components such as CRISPR-Cas to cells to correct a mutation or to regulate a gene’s expression. Gene delivery vectors can either be viral or non-viral. The former harnesses viruses’ natural ability to infect host cells with their genome and are genetically modified to remove pathogenic genes and replace them with the sequence of interest. The most commonly used viral vectors are lentiviruses and adeno-associated viruses (AAVs). Lentiviruses, a subspecies of retroviruses, integrate their cargo into the host’s genome, assuring long-term expression of the delivered gene, but carrying the risk of random insertional mutagenesis. While AAVs offer a rather limited carriage capacity (~4.5 kb), they are deemed large enough for most genes used in therapy. They are also considered a much safer alternative because their genomes exist as an independent episome in the transfected cell, and they are also capable of conferring long-term expression in non-dividing neurons [66,67]. Furthermore, the use of certain serotypes (e.g., AAV9) carries certain advantages, such the ability to cross the BBB [68], a major obstacle in the way of vectors to the brain.

Conventional gene therapy utilizes viral vectors for the delivery of therapeutic transgenes. The first viral-mediated gene therapy was approved for clinical use in 1990 for adenosine deaminase-severe combined immunodeficiency (ADA-SCID). In it, two young girls were treated with autologous T-lymphocytes genetically modified with retroviruses carrying a wild-type ADA gene ex vivo [69,70]. Evidence of modest feasibility encouraged subsequent gene therapy trials to take place, to which major limitations ensued. For example, gene therapy trials for SCID resulted in the development of therapy-related leukemia in several young children, leading to the death of one patient [71,72]. Investigations of the possible cause of leukemia revealed insertional mutagenesis of the therapeutic gene into a proto-oncogene locus [73]. Consequently, various bodies modeled by the US Recombinant DNA Advisory Committee and the Food and Drug Administration have been established to acknowledge this risk and to standardize protocols for gene therapy in humans globally [74].

Non-viral vectors are synthetic, which gives them a unique flexibility to be customized for use from a range of different compounds, such as lipids and proteins. The components of synthetic vectors are inspired from the composition of viral envelopes that enable viruses to specifically bind to host cells. However, they offer a safer alternative to viral vectors with possibly lesser mutagenic and immunogenic responses in the host [75]. It has been shown that by integrating a fragment derived from the rabies virus glycoprotein (RVG), these vectors can be modified to cross the BBB and deliver their cargo to specific neuronal cells for the treatment of PD [76,77]. Thus far, all gene therapy clinical trials for PD have been carried out using viral vectors, since non-viral vectors need to be optimized to provide equal delivery efficiency to viral vectors.

Gene editing approaches, such as using CRISPR, are capable of inducing genetic insertions and corrections at a specific locus and offer an alternative to introducing a therapeutic gene into a random genomic locus. The emergence of such approaches further helps to overcome the critical limitation of insertional mutagenesis.

### 2.1. Gene Therapy Trials for PD

Most gene therapy clinical trials have fulfilled Phase I, safety and efficacy profiles, but the majority failed when advanced to controlled, blinded Phase II trials to achieve results beyond placebo effect or better than those seen with current treatments (Table 1). Importantly, these studies represent a proof of concept of how PD can be tackled at the genetic level. Ongoing trials focus either on symptomatic benefit through balancing physiological basal ganglia circuitry or enhancing the dopamine biogenesis pathway, or on disease modification through providing neuronal protection or preventing α-synuclein aggregation or accumulation. The principles, progress and results of these trials are discussed below to highlight how gene therapy could be used to tackle PD on these different levels, emphasizing its broad scope of intervention.

#### 2.1.1. Restoring the Physiological Balance of the Basal Ganglia

The loss of dopaminergic neurons in PD leads to a hyperactive STN, which can be reversed by injections of an agonist of γ-aminobutyric acid (GABA), the inhibitory neurotransmitter of the STN, as shown previously in non-human primate (NHP) PD models [89].

##### GABA

PD-like symptoms were alleviated in rat and NHP models via overexpression of glutamic acid decarboxylase (GAD), the enzyme involved in the synthesis of GABA, in the STN [90,91]. Two genetically distinct GAD isoforms, GAD65 and GAD67, were both used in a trial to increase GAD expression in the STN [78]. The trial’s aim was to increase GABA production and the inhibition of the STN, thus attaining the same effect of DBS and improving motor deficits. In Phase I, all patients who received a unilateral AAV2-GAD injection to the STN showed improvements on their unified Parkinson’s disease rating scale (UPDRS) scores which persisted throughout the 12-month duration of the study [78]. Subsequent positron emission tomography (PET) scans using [^18^F] fluoro-deoxyglucose as a tracer showed a significant reduction in glucose uptake in the thalamus of the treated side, indicating a reduction in thalamic metabolic activity, in line with the improved motor functions [78]. In a double-blinded Phase II trial, patients who received AAV2-GAD injection had improvements in UPDRS scores over the sham control group [79]. Although gene therapy ameliorated PD symptoms in these trials with no adverse events reported in any of the patients and effects persisting for a year [80], they showed no greater improvement over current standards of care. It is important to note that these studies serve as a proof-of-principle approach for a generally safe and efficacious operation for gene therapy which is valuable for optimizing the design of larger clinical trials in the future.

#### 2.1.2. Enhancing Dopamine Synthesis

Dopamine is synthesized in dopaminergic neurons from the amino acid tyrosine derived through diet. Tyrosine is first converted to L-DOPA by tyrosine hydroxylase (TH), and aromatic amino acid decarboxylase (AADC) converts L-DOPA to dopamine (Figure 1C). Guanosine triphosphate cyclohydroxylase I (GCH1) is the rate-limiting enzyme in synthesizing the TH co-factor tetrahydrobiopterine (BH4). These enzymes are transported from the SNc to the striatum through the nigrostriatal pathway in an anterograde manner (Figure 1D). In advanced PD, severe dopaminergic nerve loss is associated with a significant reduction in the activity of these enzymes in the striatum. In addition, as the disease progresses, L-DOPA dosage requirements for patients increase and the resulting elevated levels of dopamine outside the basal ganglia lead to dyskinesia.

##### *AADC* 

Gene transfer of AADC has been used to enhance the pathway of dopamine biogenesis, by rescuing AADC levels dropping with the degeneration of nigral dopaminergic neurons [81,92,93,94,95,96,97]. After assessment in animal models [92,93,94], Phase I clinical trials using AAV2 [81,95,96] concluded that AAV-AADC gene transfer is safe and stable and patients showed clinical improvements in the first 12 months; however, this improvement slowly deteriorated. This was attributed to the restricted distribution of AADC expression, and the relatively small final volume of vector infused. Consequently, researchers have developed a novel mechanism of delivery, with the aim of increasing the coverage of the striatum, using an MRI-guided convection enhanced delivery (CED) to monitor the delivery of the transgene in non-human primates in real time [97]. Accurate positioning of the cannula was confirmed by MRI images indicating increased coverage of the targeted affected mid-brain neurons in all animals. Further assessment by immunohistochemical staining confirmed the increased expression of AADC in the SNc compared to control animals, which correlated with an increase in AADC concentration in the striatum, indicating successful axonal transport throughout the nigrostriatal pathway. Indeed, as this trial has achieved safety and efficacy and an MRI-CED delivery of AAV2-AADC, it should be assessed for efficiency in a human clinical trial.

##### *TH*, *AADC* and *GCH1*

A lentiviral-vector-based gene therapy aimed at increasing the efficiency of patients’ metabolism of L-DOPA, and thus decreasing the dose of L-DOPA required and avoiding treatment-related side effects [85,98]. Initially, triple gene transfer of CGH1, TH and AADC in a single lentiviral transcriptional unit was first demonstrated to successfully increase dopamine production and reduce motor asymmetry in rat models of PD [98]. Recently, in a dose-escalation clinical trial, patients who received the Lenti-TH-AADC-GCH treatment demonstrated dose-dependent improvements in their UPDRS scores after 12 months, with the group receiving the highest dosage displaying the most improvement [85]. The study was extended, and patients who were still part of the study showed further improvements after 24 and 36 months [85]. Overall, the trial achieved safety and efficacy, but no control group was included, and the improvements were within the placebo effect seen in other trials [85]. This trial, however, represents the first where lentiviral vectors have been successfully applied to the treatment of a neurological disorder. Moreover, this approach does not depend on dopaminergic neurons, but rather assumes that the transfected striatal neurons will develop the ability to synthesize dopamine. However, striatal neurons lack other mechanisms intrinsic to dopaminergic neurons, such as the capacity to store synthesized dopamine in synaptic vesicles and to take it back up after release through dopamine transporters, which is perhaps why there was no significant improvement.

#### 2.1.3. Neuroprotection and Regeneration

The loss of SNc dopaminergic neurons caused by PD pathology results in the reduction in dopamine levels in the striatum. Thus, providing support and protection to dopaminergic neurons from degeneration is an attractive strategy to prevent the manifestation of downstream complications such as physiological imbalances, and symptoms such as rigidity and bradykinesia associated with the disease.

##### *GDNF*, *NRTN*

Gene therapy trials offering neuroprotection have focused primarily on the delivery of members of the glial cell family of ligands (GFLs), which are known to play a role in cell protection and survival [99]. For example, the delivery of neurturin (NRTN) to the putamen using AAV2 was well tolerated in a Phase I clinical trial [86], but no improvements in motor function were observed [87]. Analyses of postmortem tissue showed an increase in NRTN expression in the putamen; however, due to a failure of retrograde transport, it was not upregulated in the SN [100]. Moreover, no improvements in motor function were seen in a clinical trial using higher doses of AAV-NRTN to address this issue [88]. On another hand, in animal models, glial-derived neurotrophic factor (GDNF) expression protected nigrostriatal neurons and improved motor function. However, it was found that its long-term expression causes aberrant axonal sprouting and downregulation of TH [101,102]. GDNF is a growth factor that was the first GFL to be discovered, and it functions in promoting the survival of dopaminergic neurons [103]. Further studies demonstrated that injections of AAV-GDNF were well tolerated [104,105]; however, bilateral SN injections resulted in weight loss in aged monkeys [105]. Two ongoing Phase I clinical trials are currently testing the safety of bilateral injections of AAV2-GDNF into the putamen (NCT01621581 and NCT04167540). Importantly, a system whereby the expression of GDNF can be controlled after delivery was developed and showed neuroprotection and improvement in motor function in rodent models [106,107]. In this method, the destabilizing domain of *E. coli* dihydrofolate reductase was fused to GDNF and delivered to neurons using a lentiviral vector. The expression was controlled by the temporal administration of Trimethopram, a drug that crosses the BBB to stabilize the destabilizing domain and activate GDNF expression [106]. The treated group showed improved motor function and a higher level of TH expression than rodents receiving a normal GDNF transfusion. This chemical method sets an adaptable protocol using destabilizing domains to control the expression of other genes of interest. Some PD patients exhibit downregulation of Ret, the receptor for GDNF and NRTN, which could explain the lack of success seen in the above neurotrophic growth factor gene therapies [108]. Additionally, the degenerative state of the PD brain may affect the transport of growth factors, suggesting that such gene therapies may improve patients receiving therapy earlier in the disease course.

##### Neural Regeneration

An exciting gene therapy approach tackling PD is generating new neurons through converting astrocytes, abundant in the brain, into induced dopamine-releasing neurons [109,110]. Two teams showed that depletion of PTB, an RNA-binding protein which suppresses neuronal differentiation, using short hairpin RNA (shRNA) [110] or through viral delivery of CasRx, an RNA-targeting CRISPR-Cas, to the brain [109], converted resident astrocytes into neurons and rescued neurochemical and motor deficits in mice [109,110].

#### 2.1.4. Targeting Disease Genes

##### *SNCA*, *LRRK2* and *GBA*

As discussed in previous sections, evidence links mutations increasing the expression of α-synuclein to PD and to the formation of Lewy bodies associated with neurodegeneration [23,37,38,39]. It is thus thought that providing neuronal protection could be achieved through downregulating *SNCA*. Knocking down α-synuclein by shRNA or anti-sense oligonucleotides (ASOs) was reported to prevent neurodegeneration in PD models [111,112,113]. AAV-mediated delivery of shRNA-targeting endogenous *SNCA* in rats attenuated rotenone-induced progressive motor deficits and neurodegeneration [111]. Furthermore, *SNCA* downregulation ameliorated neurological deficits in mice models expressing human α-synuclein [112]. Cole et al. (2021) recently showed that ASO-mediated reduction of α-synuclein reversed PD pathology and rescued dopaminergic neuronal function in rodent models of PD [113]. Furthermore, the study reported decreased human α-synuclein levels in the cerebrospinal fluid of non-human primates as a demonstration of the translational potential of the approach [113]. Downregulation of SNCA using CRISPR was also shown to improve cell viability in a PD patient’s iPSC-derived dopaminergic neurons [114]. However, other studies found lowering SNCA levels to be associated with further pathologies in vivo, an issue which is yet to be solved [115,116]. Short interfering RNA (siRNA) delivered into rats’ brains using AAV2/5 vectors to block the translation of α-synuclein in the SNc resulted in even worse motor and behavioral deficits accompanied by reduced TH levels and nigral dopaminergic neurodegeneration [115]. This indicates that both the overexpression and downregulation of SNCA may have a negative effect on neuronal survival, and hints at essential functions of the α-synuclein protein in a healthy brain. It is necessary that alternative strategies tackling α-synuclein are explored. For example, expressing six mutant versions of α-synuclein that block the aggregation of wild-type α-synuclein has shown promising results, but has yet to be validated in vivo [117]. Using CRISPR-interference (dCas9-KRAB), Heman-Ackah et al. (2016) knocked down α-synuclein in iPSCs carrying the *SNCA* triplication mutation [118]. Another group used an epigenetic approach (dCas9-DNMT3A) by hyper-methylating *SNCA*’s intron 1 to restore normal mRNA levels in iPSC-derived dopaminergic neurons [114].

LRRK2 mutations cause familial PD or may increase the risk of developing sporadic PD [119]. G2019S and other LRRK2 mutations associated with PD lead to an increased kinase activity, and thus, knocking it out is a sound therapeutic strategy. However, studies have shown that LRRK2 depletion may lead to pathological consequences in other tissues where LRRK2 is expressed, such as the lungs, kidneys and spleen [120,121]. Alternatively, direct intracerebral injections of ASOs depleted LRRK2 protein levels, reduced fibril-induced α-synuclein inclusions and protected TH^+^ neurons in the brain of mice, with no detected pathological phenotype in other tissues [122]. A Phase I clinical trial using LRRK2 ASO intrathecal injections is currently ongoing in patients with PD (NCT03976349). However, it is essential to employ routes of delivery capable of brain-specific targeting to apply the LRRK2 gene therapy in a less invasive setting.

A potential gene therapy approach for tackling loss-of-function forms of PD is to overexpress the functional protein. A significant proportion of recessive PD is associated with loss-of-function mutations in PRKN and PINK1 genes involved in mitochondrial function and mitophagy [23,123,124,125]. However, since motor symptoms in such forms of PD are efficiently treated by levodopa, it may be unnecessary to develop a gene therapy to restore Parkin function as an alternative treatment.

Mutations in GBA cause Gaucher’s disease, a lysosomal storage disorder, and also represent the most common risk factor for developing PD [126]. A study reported that direct AAV-GBA1 injections in the brains of rodent models of PD reduced α-synuclein levels and pathology [127]. Moreover, intravenous injections of AAV-PHP.B-GBA1 alleviated α-synuclein pathology and produced significant behavioral recovery in A53T mouse models of PD [128]. Intracisternal injection of AAV9-GBA1 to treat PD patients is currently undergoing a Phase I/II clinical trial (NCT04127578).

## 3. Prospective and Conclusive Remarks

Many genes have been implicated in the pathogenesis of PD. It is reasonable to think of SNCA as a target for gene therapy to prevent the toxic accumulation of α-synuclein. It was shown that downregulating SNCA is associated with further neurodegeneration and pathogenesis [103]. Other results suggest that expressing a mutant form of α-synuclein may be an efficient strategy in blocking its aggregation [106]. Correcting the SNCA mutation or replacing it with a normal allele may also prove effective against the accelerated protein misfolding caused by elevated or aberrant SNCA expression. Indeed, there is a lack of reports of gene correction for PD, and it would be interesting to assess whether it is sufficient to alleviate the neuropathology caused by DNA mutations in general. However, it is important to note that such approaches do not account for potential environmental factors or additional genetic susceptibilities that may have a higher contribution to the α-synuclein pathology. Since mutations in GBA and lower levels of GCase have been associated with Lewy body pathology [85,92], gene correction may serve as a therapeutic strategy for reducing α-synuclein accumulation. Other factors than direct mutations in GBA may be the cause of reduced levels of this protein, and overexpressing GBA to dispose of Lewy bodies can therefore be an effective disease-modifying strategy for neuroprotection.

PD is considered a synucleinopathy, and more results continue to point towards the direction of α-synuclein aggregation as the cause of neuronal cell death and impairment. Increasing evidence suggests that cellular degradation pathways, such as CMA, may be responsible for the toxic accumulation of α-synuclein. Regulating lysosomal degradation puts forth a potential in treating PD or any other synucleinopathy where abnormal levels of α-synuclein are the cause of neurodegeneration. However, it is worth noting that CMA is an essential mechanism of cellular lysosomal degradation which is not exclusive to α-synuclein. Therefore, enhancing it would subsequently accelerate the degradation of other cellular components possibly essential for cell survival. Thus, more in-depth in vivo studies are urgently required to reveal the possible side effects of enhancing such pathways on cell viability.

Most gene therapy trials for PD serve as a proof of concept for the safety and tolerability of different approaches tackling the disease. Importantly, they showcase the broad capability of gene therapy in addressing all levels of PD pathogenesis. They included strategies to restore dopamine synthesis, enhance neuroprotection, enhance lysosomal and degradation pathways and restore the physiological balance of the basal ganglia. Although limited success has been reported so far, results indicate that enhancing certain parameters such as the route of delivery and infusion volumes to increase the level of transduction, dosing and tissue specificity may greatly improve the outcome. Improved neurosurgical techniques such as MRI-guided CEDs allows more accurate positioning of the cannula and control over the rate of infusion to ensure precise and homogenous infusion into the target structure. Moreover, due to the nature of the disease, early intervention is key to maximize the level of disease modification and therapy. Thus, there is a lot of promise and anticipation for the development of methods for early detection and diagnosis of PD.

Although gene therapy faced a lot of challenges in its early years, it is now considered safe for clinical use due to increased safety measures and advancements in vector development and gene editing technologies. With the increasing knowledge about pathways leading to PD pathology, gene therapy now represents an efficient disease-modifying tool for inherited and sporadic forms of the disease. The introduction of precise genome engineering tools such as CRISPR-Cas eradicates the risks associated with gene therapy, such as insertional mutagenesis or uncontrolled gene expression. Many challenges still remain and need to be addressed, such as the development of efficient delivery vectors and extensive testing to achieve adequate safety using genome editing tools.

## Figures and Tables

**Table 1:** Current status of gene therapies for Parkinson’s disease.

	Mode of Delivery	Phase	Duration	Primary Endpoint	Outcome(s)	Reference(s)
Restoring the physiological balance of the basal ganglia						
GABA						
AAV2-*GAD*	IP (subthalamic nucleus)	I	2003–2005	Safety	UPDRS improvement persisted for 12 months, and reduced thalamic activity as assessed by ^18^F-FDG PET in all patients.	NCT00195143 [78]
AAV2-*GAD*	IP (subthalamic nucleus)	II	2008–2010	Disease severity and progression	UPDRS improvement over sham control group, diminished PD symptoms with no adverse events for 12 months in all patients. No improvement over conventional PD treatment.	NCT00643890 [79,80]
Enhancing dopamine synthesis						
*AADC*						
AAV2-*AADC*	IP (striatum)	I	2004–2013	Safety and tolerability	Clinical improvements in first 12 months in all patients followed by slow deterioration. One symptomatic and two asymptomatic intracranial hemorrhages followed. Increased ON time and reduced OFF time. Increased AADC activity as measured by ^18^FMT PET.	NCT00229736 [81]
AAV2-*AADC*	IP (striatum)	I	2013–2020	Safety and tolerability	Stable increase in AADC activity as measured by ^18^F-Levodopa PET at 6 months, and clinical improvements at 12 months with no serious adverse events in all patients. Improvements were stable or improved at 12, 24 and 36 months.	NCT01973543 [82,83]
AAV2-*AADC*	IP (putamen)	I/II	2015–2018	Safety	N/A	NCT02418598
AAV2-*AADC*	IP (striatum)	I	2017–2021	Safety and suicide risk	Increase in AADC activity as measured by PET and increase in ON time without troublesome dyskinesia.	NCT03065192
AAV2-*AADC*	IP (striatum)	II	2018–2022	Change in ON time without troublesome dyskinesia	N/A	NCT03562494 [84]
*AADC, TH, CGH1*						
Lentivirus-*TH/AADC/CGH1*	IP (striatum)	I/II	2008–2012	Safety	Significant UPDRS improvement at 6 months. Mild to moderate but no serious drug-related adverse events were reported during the first 12 months.	NCT00627588 [85]
Lentivirus-TH/AADC/CGH1	IP (striatum)	I/II	2011–2022	Long-term safety and tolerability	N/A	NCT01856439 [85]
Neuroprotection						
*GDNF*						
AAV2-*GDNF*	IP (putamen)	I	2013–2022	Safety and tolerability	Stable motor scores throughout study period. Increase in ^18^F-DOPA uptake as assessed by PET at 6 and 18 months in 10/13 and 12/13 patients, respectively.	NCT01621581 [84]
AAV2-*GDNF*	IP (putamen)	I	2020–2026	Safety and tolerability	N/A	NCT04167540
*NRTN*						
AAV2-*NTN*	IP (putamen)	I	2005–2007	Safety and tolerability	Improvement of 14 points in off-medication motor score of UPDRS and increase of 2.3 h in ON time without troublesome dyskinesia at 12 months. Non-significant improvements in several secondary measures. No change in ^18^F-levodopa uptake as assessed by PET	NCT00252850 [86]
AAV2-*NTN*	IP (putamen)	II	2006–2008	Disease severity and progression	No significant improvement in UPDRS over sham surgery control group at 12 months. Serious adverse events in 13/38 treated and in 4/20 control individuals. Three patients in the treated group and two in the sham surgery group developed tumors.	NCT00400634 [87]
AAV2-*NTN*	IP (substantia nigra + putamen)	I/II	2009–2017	Disease severity and progression	No serious adverse events in all patients.	NCT00985517 [88]
Targeting disease genes						
AAV9-*Gcase*	IC	I/II	2020–2027	Safety and immunogenicity	N/A	NCT04127578

GABA: gamma-aminobutyric acid; GAD: glutamic acid decarboxylase; IP: intraparenchymal; UPDRS: Unified Parkinson’s Disease Rating Scale; FDG: fluorodeoxyglucose; PET: positron emission tomography; PD: Parkinson’s disease; AADC: aromatic l-amino acid decarboxylase; TH: tyrosine hydroxylase; CGH1: cyclohydroxylase; GDNF: glial-derived neurotrophic factor; DOPA: dihydroxyphenylalnine; NRTN/NTN: neurturin; Gcase: glucocerebrocidase; IC: intracranial.
